# Green synthesis of cellulose nanocrystal/ZnO bio-nanocomposites exerting antibacterial activity and downregulating virulence toxigenic genes of food-poisoning bacteria

**DOI:** 10.1038/s41598-022-21087-6

**Published:** 2022-10-07

**Authors:** Ghada E. Dawwam, Mona T. Al-Shemy, Azza S. El-Demerdash

**Affiliations:** 1 Botany and Microbiology Department, Faculty of Science, Benha University, Benha, Egypt P.O. 13518,; 2 Cellulose and Paper Department, National Research Centre, 33 El-Bohouth St. (Former El-Tahrir St.), Dokki, Giza, P.O. 12622 Egypt; 3 Microbiology Department, Zagazig Branch, Animal Health Research Institute (AHRI), Agriculture Research Centre (ARC), Zagazig, Egypt P.O. 4516

**Keywords:** Nanoscience and technology, Nanoscale materials

## Abstract

Recently, cellulose nanocrystals (CNs) have attracted wide attention owing to their superior properties compared to their bulk materials. For example, they represent an outstanding model for fabricating green metallic/metal oxide nanoparticles (NPs). In this study, two CNs (carboxylated CNs and sulfated CNs) extracted from agro-wastes of palm sheath fibers were used as templates for the facile and green synthesis of ZnO NPs by employing the sono-co-precipitation method. The obtained nanomaterials were characterized using TEM, EDX, UV–visible, DLS, FT-IR, and XRD analysis. As a result, the size and concentration of synthesized ZnO NPs were inversely proportional to one another and were affected by the CNs utilized and the reaction temperature used. Contagious diseases incited by multifarious toxigenic bacteria present severe threats to human health. The fabricated bio-nanocomposites were evaluated in terms of their antimicrobial efficacy by agar well diffusion method and broth microdilution assay, showing that CN–ZnO bio-nanocomposites were effective against the tested Gram-negative (*Escherichia coli* and *Salmonella*) and Gram-positive (*Listeria monocytogenes* and *Staphylococcus aureus*) bacteria. The influence of the subinhibitory concentrations of these suspensions on the expression of the most critical virulence toxin genes of the tested strains was effective. Significant downregulation levels were observed through toxigenic operons to both fabricated CN–ZnO bio-nanocomposites with a fold change ranging from 0.004 to 0.510, revealing a decline in the capacity and virulence of microorganisms to pose infections. Therefore, these newly fabricated CNS–ZnO bio-nanocomposites could be employed rationally in food systems as a novel preservative to inhibit microbial growth and repress the synthesis of exotoxins.

## Introduction

The agricultural sector all over the world, especially in developing countries, plays an important role in economic development. This agricultural sector generates a large amount of waste every year. Incineration is the most common way to dispose of this waste, which leads to environmental pollution and waste of great national wealth. That is why a major concern since the past era has been the exploitation of this renewable waste in environmentally safe ways and its transformation into new valuable products^1^. For example, the total agricultural waste produced each year in Egypt was estimated to be 30 million tons/year^2^. Cellulose, as a component of agricultural waste, has great importance and economic return in many diverse fields. Cellulose is among the most renewable, sustainable, and affordable resources that have drawn considerable attention, particularly in the form of nanocellulose^3,4^. The use of cellulose nanocrystals (CNs) in several sectors, including bio-nanocomposites, thin films, membranes, biosensors, and self-healing, has increased recently. CNs bio-nanocomposites are exceptional in many ways, such as their excellent safety profile, biocompatibility, biodegradability, and availability, as well as the accessibility and affordability of their pristine materials^5,6^. The increase in surface functional groups, notably OH groups, adsorption capacity, and mechanical strength are all benefits of the conversion of cellulose to nanoscale structures. As a result, the cellulose surface's nano-properties enable strong binding of inorganic substances like metal oxide nanoparticles (NPs), facilitating the excellent permanence of nanomaterials^7–9^.

The isolation of CNs from various natural cellulosic materials occurs in two stages. (1) The pretreatment process aims to completely or partially remove wax, hemicellulose, holocellulose, and lignin. (2) The controlled enzymatic treatment or chemical hydrolysis can be used to extract high-crystallinity CNs by removing amorphous regions^10^. After the controlled hydrolysis of cellulose (chemical or enzymatic), CNs with highly crystalline rigid rod-like hydrophilic particles that show a typical whisker structure and monocrystalline domains with diameters of 1–100 nm and lengths of the order of 100 nm to hundreds of nanometers are obtained^11^. The whiskers are 100% cellulose and show high crystallinity (*CrI* = 54%–88%). CNs are formed by the splitting of cellulose fibers and the hydrolysis of the amorphous regions. The extraction of CNs based on chemical treatments using strong acids (e.g., hydrochloric, phosphoric, and sulfuric acids) has been successfully performed in hydrolyzing cellulose fibers^4^. Regarding the final properties of CNs, the literature shows that CNs can be employed in food packaging applications because their durability features are considerably higher than those of diverse commercially available polymeric and metallic products^12^. The remarkable properties of CNs allow their use as a reinforcement phase in many other applications (e.g., thermoplastic and/or thermosetting matrices). Moreover, owing to their biocompatible and nontoxic nature toward cells, they can be used in biomedical applications for fabricating medical devices^13^. Recently, compared with its potassium and sodium counterparts, ammonium persulfate (APS) has attracted attention because of its ideal properties for CNs extraction, such as low-cost, high-water solubility, and low long-term toxicity. Therefore, as a green strong oxidant, extraction of APS is used instead of acid hydrolysis to produce CNs with enhanced homogeneity. This versatile one-pot procedure involving the use of APS can process diverse cellulosic fibers without any pretreatment to hydrolyze the other plant contents (e.g., hemicellulose and lignin). The employ of APS affords highly carboxylated CNs, as opposed to sulfated CNs produced using sulfuric acid^14,15^.

Large-scale manufacturing of NPs free of contaminants is possible thanks to green synthesis, which uses various biological entities. Many of the negative impacts of physical and chemical processes can be avoided through the green synthesis of NPs using plants, algae, fungi, actinomycetes, and bacteria^6,16,17^ These involve producing NPs naturally under benign conditions of temperature, pH, and pressure, without the use of hazardous or toxic materials, and without the need to add external reducing, capping, or stabilizing agents^18–20^. Cellulose nanofibers, CNs, or bacterial cellulose, may function as a polymer base to augment the efficacy of metal/metal oxide NPs. The consequential hybrids are worthy substances that exhibit novel antibacterial, magnetic, electronic, and optical properties, thus showing potential in biomedical applications^21^. As antibacterial formulations, nanocellulose–metal oxides are preferred over antibiotics because they lack microbial impedance, which is the major reason for the failure of antibiotics in healing infectious diseases^21^. In food engineering applications, ZnO NPs have garnered considerable attention because they are stable and robust and show intrinsic antimicrobial properties and a long shelf life^22,23^ in addition to their safety for human consumption^24^. When surveying the literature that dealt with the preparation of ZnO NPs, we found many of them dealt with the synthesis of ZnO NPs using chemicals^25,26^, calcination^27^ or ,plant extracts^28–31^. Even those who studied the bio-nanocomposites of CNs with ZnO NPs either prepared ZnO NPs first and then impregnated them on CNs^21,32,33^ or their precursors were chemically reduced by NaOH in the presence of CNs^34–36^. As far as we know, ZnO NPs have not been synthesized before by exploiting the functional groups on the CNs with the help of ultrasonic waves only.

One of the most important aspects that represent a threat to the health of consumers, especially in developing countries, is foodborne diseases. Most foodborne diseases are caused by pathogenic bacteria belonging to the genera *Escherichia*, *Listeria*, *Salmonella*, and *Staphylococcus*^37^. In general, bacterial pathogens induce diseases that can usually be treated with antibiotics^38,39^. These antibiotics possess bactericidal modes that hinder the development of bacterial pathogens via different routes of activity, varying from protein, RNA, and DNA synthesis interference to enzyme action suppression^40^. However, bacterial strains can show antimicrobial resistance to such antibiotics owing to (1) the formation of enzymes that can degrade or modify the drug^38,41^; (2) modification of the base of antibiotics by describing genes that character a substitute type of the antibiotic base^41,42^; (3) function as propels pumps that force out the drug or decrease the adsorption of antimicrobial drugs^40^; (4) colonization and production of toxins^43^; and (5) creation of biofilm layers surrounding the bacterial cell to restrict or lower its vulnerability to antibiotics^44^. Thus, the issue of increased bacterial resistance to antimicrobial compounds constitutes a real threat to human health, necessitating the development of effective agents and strategies.

Currently, the layout of antimicrobial or antibacterial substances involves various substantial factors, such as the utilization of food or agricultural waste and affordable raw materials, processing method and facile synthesis, biocompatible characteristics for food or medical implementations, and fabrication of biodegradable products that reduce the environmental consequence at the end of the life span^45^. *Escherichia coli* and *Staphylococcus aureus*, the classic microorganisms of Gram-negative and Gram-positive bacteria, can adhere to surfaces, as well as colonize and form biofilms on them. These bacterial species are the criterion microbial indices of food pollution in food manufacture, and their restrain implies the satisfactory hygiene of surfaces and processing materials in food industrialization^46^. Another common foodborne pathogen microorganism with the potential to form biofilms and to live on diverse sorts of surfaces even under unfavorable conditions, such as high salt concentrations, dry environments, low pH, and low temperatures, is *Listeria monocytogenes*^47,48^. Moreover, *Salmonella* causes most foodborne diseases^37^.

In this study, two CNs extracted from palm sheath fibers were used as templates for the facile and green synthesis of ZnO NPs by employing the sono-co-precipitation method. The obtained CNs and CNS–ZnO bio-nanocomposites were characterized using TEM, EDX, UV–Vis, DLS, FT-IR, and XRD analysis. We believe that these biosynthesized metal–oxide NPs have shown worthwhile results when used as therapeutic agents contra some infectious diseases induced by multidrug-resistant Gram-positive and Gram-negative bacteria. Moreover, the effect of the newly prepared CN–ZnO bio-nanocomposites on reducing virulence and the ability of toxigenic bacteria to cause infection has not been studied before which makes it the focus of many applications, especially in food packaging. As a result, the goal of this work is to evaluate the effectiveness of environmentally friendly CN–ZnO bio-nanocomposites and in showcasing their prospective use as antibacterial agents with anti-toxigenic properties. Further, the current research is investigating these synthesized CN–ZnO bio-nanocomposites' capacity to inhibit key genes encoding major modulatory toxins from the pathogenic bacteria under study.

## Experimental method

### Materials

Palm (*Phoenix dactylifera* L.) sheath fibers (PSFs) were collected after pruning in the fall of 2019 from Sohag Governorate in Upper Egypt. All chemicals used in this study were of analytical grade and used as supplied without any further purification. APS and nutrient agar media were obtained from Sigma-Aldrich. Sulfuric acid (97%) and zinc acetate dihydrate were procured from Abco Chemie (ENG. Ltd.) and Laboratory Rasayan, respectively. The DNA extraction of samples was performed using a QIAamp DNA minikit and a 2 × QuantiTect SYBR Green PCR Master Mix (both from Qiagen GmbH, Germany).

### Sample preparation

#### Isolation of CNs

First, the PSFs were subjected to successive treatment processes of pulping and bleaching. The pulping process was performed using 17.5% (w/w) sodium hydroxide and liquor ratio (1:7) at 160 °C for 90 min. Thereafter, the bleaching treatment was carried out three successive times with 3.5% sodium chloride at 70 °C and pH of 3 for 1 h for each step. Each stage of the processing treatments, i.e., pulping and bleaching, was pursued by scrubbing with sufficient distilled water to neutral pH. Second, the CNs were obtained through two different extraction processes. CNAs were extracted from the unbleached pulp of a PSF using 1.25 M APS at 70 °C, 1:100 liquor ratio, and 16 h, whereas CNSs were extracted from the bleached pulp of a PSF using 6.5 M sulfuric acid at 50 °C, 1:20 liquor ratio, and 2 h^4,49^. The resultant suspensions from each extraction process were centrifuged at 15,000 rpm for 15 min, pursued by dialysis versus distilled water until the pH reached 5. At last, before additional treatments and characterizations, the extracted CNs (CNAs and CNSs) were lyophilized.

#### Fabrication of the CNA–ZnO, and CNS–ZnO bio-nanocomposites

**Figure 1:**
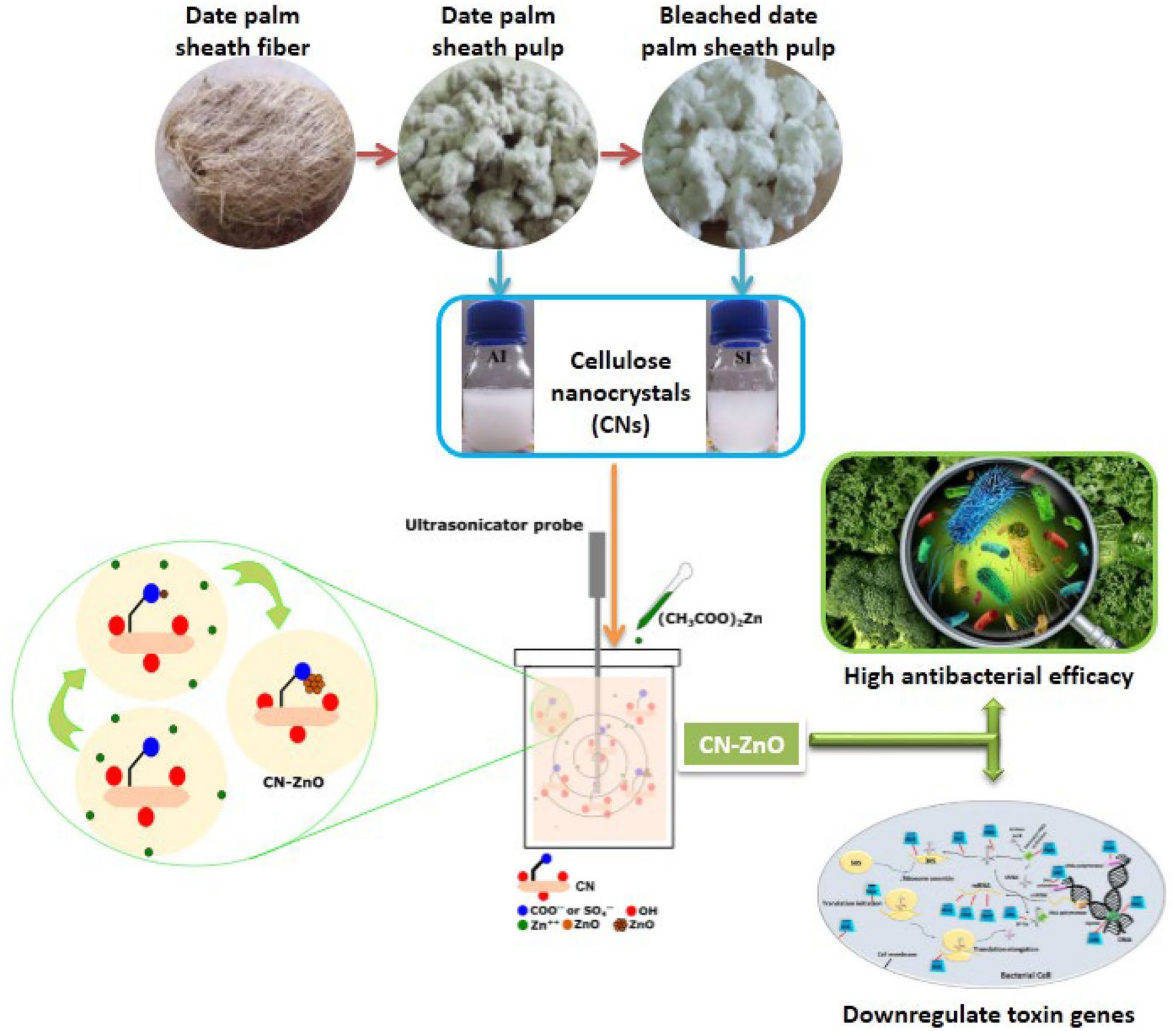
Graphical abstract of the fabrications and applications of nanomaterials under investigation.

CN–ZnO bio-nanocomposites were prepared via a sono-coprecipitation method using CNAs and CNSs at 80 °C and 35 °C, respectively. In detail, 3.5 g of zinc acetate (a precursor of ZnO) was added to a 1% CNAs suspension. The solution was stirred for 30 min before being sonicated at a temperature of 80 °C for 2 h. The same procedure was applied to CNSs at 35 °C. The resultant white precipitates were dialyzed against deionized water to remove unreacted salts and impurities. The washing process continued until the dialyzing water conductivity became ≈ 4 μS·cm^−1^. Finally, the resulting slurries were dried in an oven for 6 h at 60 °C on Teflon plates to yield CN–ZnO bio-nanocomposites^50^. The obtained CNs–ZNO bio-nanocomposites white precipitates were then packaged for subsequent characterizations and applications. An overview of the work done in this study is depicted in Fig. 1.

### Analytical characterization

#### UV–visible (UV–Vis) spectroscopy

The optical characteristics of the fabricated CNAs, CNSs, CNA–ZnO, and CNS–ZnO aqueous suspensions were investigated using UV–Vis spectroscopy within the range of 200–800 nm using Agilent’s Cary 50 probe equipment.

#### Fourier-transform infrared (FT-IR) spectroscopy

The FT-IR spectra of the differently prepared samples were measured on a JASCO FT-IR 6100 spectrometer (Tokyo, Japan). The records were conducted in the range of 400–4000 cm^−1^ with a resolution of 4 cm^−1^ and 32 scans.

#### X-ray diffraction (XRD) spectroscopy

The XRD patterns of the prepared lyophilized CNAs, CNSs, CNA–ZnO, and CNS–ZnO were analyzed using a Malvern Panalytical Empyrean X-ray diffractometer (the Netherlands), with an angle of incident monochromatic X-ray (2*θ*) within the range of 5°–70°. The crystallinity index (*CrI*) was predicted using the Segal formula:

$$CrI\left(\mathrm{\%}\right)= \left[\frac{{(I}_{200}-{I}_{\mathrm{am}})}{{I}_{200}}\right]\times 100$$

here *I*_200_ denotes the overall intensity of the lattice peak at 2*θ* ≈ 22.6°, and *I*_am_ represents the intensity of the amorphous domain at 2*θ* ≈ 18°, where the intensity is the lowest^51^. The crystallite size (*L*_200_) was evaluated using the Scherrer formula^52^:

$${L}_{200}(\mathrm{nm})= \frac{K\lambda }{\beta \mathrm{cos}\theta }$$

Moreover, the Bragg’s law was used to calculate *d-spacing* (Å) between the crystal planes:

$$d-spacing= \frac{n\lambda }{2\mathrm{sin}\theta }$$

here *K* denotes a constant with a value of 0.94; *λ* represents the X-ray wavelength; *β* denotes the half-height width of the diffraction peak; *n* represents an integral number (1, 2, 3, …); and *θ* denotes the Bragg angle corresponding to the (200) lattice plane in the case of CNs.

#### Morphological analysis

A Quanta FEG-250 (Waltham, MA, USA) scanning electron microscope at a voltage of 20 kV was used for depicting and analyzing elements via the energy-dispersive X-ray spectroscopy (EDS) of dry CNA–ZnO and CNS–ZnO bio-nanocomposites. Moreover, transmission electron microscopy (TEM) micrographs were captured with high-resolution JEOL JEM-2100 (Japan). The prepared suspensions were diluted 10 times before being dried on a microgrid coated by a thin carbon film (≈ 200 nm). In contrast to the dried residues of CNA–ZnO and CNS–ZnO nanocomposites, the dried precipitates of CNAs and CNSs were stained with a 2% uranyl acetate solution to improve the microscopic resolution.

#### Average size and Zeta-potential analysis

Using the high concentration zeta potential cell ZEN1010, zeta-potential and average size evaluations of the produced CNA and CNS were performed on a Malvern Nano Zeta-sizer (Malvern, NanoZS, UK) at 200.1 °C. Across the nominal electrode spacing of 16 mm, a 40 V field was applied. Before the experiments, the samples were ultrasonicated for 5 min at 50% amplitude to improve particle dispersibility.

### Antimicrobial activity

#### Collection of microbial pathogens

Four different strains of each pathogenic multidrug-resistant bacterium such as *E. coli* (ATCC 25922, ATCC 8739, ATCC 35218, ATCC 51755), *Salmonella *(*Salmonella Typhimurium* ATCC 14028, *Salmonella Choleraesuis* ATCC 10708, *Salmonella Montevideo ATCC* 8387, *Salmonella Enteritidis* ATCC 13076*)*, *L. monocytogenes *(ATCC 7646, ATCC 7644, ATCC BAA-751, ATCC 19155) and *S. aureus *(ATCC 6538, ATCC 29737, ATCC 29213, ATCC 43300) were utilized in the current study.

#### Antibacterial potential assay

##### Determination of the antibacterial efficacy of bio-nanocomposites

The antibacterial efficacy of the CNA–ZnO, and CNS–ZO bio-nanocomposites was tested against Gram-positive (*L. monocytogenes* and *S. aureus*) and Gram-negative (*E. coli* and *Salmonella*) bacteria employing the agar diffusion approach according to Khosravi et al.^53^. The bacteria were grown in a nutrient liquid medium on a shaker bed at 200 rpm for 24 h at 37 °C. The bacteria (1.5 × 10^8^ CFU) were swabbed on nutrient agar plates; subsequently, 200 µL of orthogonal array samples were deposited in wells (diameter of 7 mm) molded in the agar plates, and those plates were cultured for 24 h at 37 °C. Further, the dimensions of the inhibition zone (i.e., clear areas) were measured. All samples were measured in triplicates.

##### Minimum inhibitory concentration (MIC) and minimum bactericidal concentration (MBC) measurement

The broth microdilution process was employed with 96-well plates (TPP, Switzerland). Each extract was diluted twofold in LB broth® (Acumedia, Michigan, USA), and the wells were injected with 1 × 10^6^ CFU of bacteria (in a 0.2 mL final volume). After incubation at 37 °C for 24 h, the MIC analysis was implemented consistent with the references of the Clinical and Laboratory Standards Institute^54^. The extent of concentrations investigated for each suspension ranged from 0.062 to 64 µg/mL. The MIC value is defined as the lowest antimicrobial concentration that impedes the development of microorganisms, while the SIC value is defined as the antimicrobial concentration less than one capable of impeding the observed development and reproduction of the microorganism.

### Molecular assay

#### DNA extraction and PCR amplification

The QIAamp DNA mini kit (Qiagen GmbH, Germany) was utilized for the extraction of DNA from samples. Further, strains were examined for the existence of toxin virulence parameters, as shown in Table 1.

**Table 1:** Different primers and their sequences of different bacteria used for detecting the toxin virulence factors.

Microorganism	Primer	Sequence	Amplicon size	References
*E. coli*	*stx1*	ACACTGGATGATCTCAGTG CTGAATCCCCCTCCATTATG	614	^55^
	*stx2*	CCATGACAACGGACAGCAGTT CCTGTCAACTGAGCAGCACTTTG	779	
*Salmonella* spp*.*	*stn*	CTT TGG TCG TAA AAT AAG GCG TGC CCA AAG CAG AGA GAT TC	260	^56^
*L. monocytogenes*	*hlyA*	CCT AAG ACG CCA ATC GAA AAG CGC TTG CAA CTG CTC	702	^57^
*S. aureus*	*sea*	TTGGAAACGGTTAAAACGAA GAACCTTCCCATCAAAAACA	120	^58^
	*seb*	TCGCATCAAACTGACAAACG GCAGGTACTCTATAAGTGCC	478	
	*sec*	GACATAAAAGCTAGGAATTT AAATCGGATTAACATTATCC	257	
	*sed*	CTAGTTTGGTAATATCTCCT TAATGCTATATCTTATAGGG	317	
	*see*	TAGATAAAGTTAAAACAAGC TAACTTACCGTGGACCCTTC	170	

#### PCR product visualization and examination

The products of PCR were separated via electrophoresis on 1% agarose gel (AppliChem GmbH, Germany) by running 20 μL of the PCR products. The gel was photographed using a gel documentation system (Alpha Innotech, Biometra), and the data were analyzed via computer software.

#### Quantitative analysis of the toxin gene expression

Quantitative real-time PCR (qRT-PCR) was utilized for the analysis of toxin gene expression whereas the 16S rRNA housekeeping gene of each pathogen functioned like an interior control to standardize the expressional ranks among samples. Primers were employed in a 25 μL reaction comprising 3 μL of the RNA template, 8.25 μL of water, 0.5 μL of each primer of 20 pmol concentration, 0.25 μL of RevertAid Reverse Transcriptase (200 U/μL) (Thermo Fisher), and 12.5 μL of the 2 × QuantiTect SYBR Green PCR Master Mix (Qiagen GmbH, Germany). The reaction was implemented in step one real-time PCR under precise conditions stated in Table 2. Ct values and amplification curves were estimated. To evaluate the divergence in gene expression on the RNA of the various samples, following the “ΔΔCt” procedure presented by Yuan et al. the Ct of each sample was analogized with that of the positive control group^59^.

**Table 2:** Objective genes and cycling conditions for SYBR green Rt-PCR.

Objective gene	Reverse transcription	Primary denaturation	Amplification (40 cycles)			Reference
			Secondary denaturation	Annealing (optics on)	Extension	
*E .coli 16S RNA* *stx1* *stx2*	30 min. 50 °C	15 min. 94 °C	15 s. 94 °C	30 s. 55 °C	30 s. 72 °C	^60^
*Salmonella* 16S RNA *Stn*				1 min. 60 °C		^61^
*L. monocytogenes* 16S RNA *hlyA*				30 s. 49 °C		^62^
*S. aureus* 16S rRNA *sea* *seb* *sec* *sed* *see*				1 min. 55 °C		^63^

### Statistical analysis

Results were expressed as mean ± standard deviation and the data were analyzed using one-way ANOVA using GraphPad Prism for windows, http://www.graphpad.com. The differences between means were detected by Tukey’s test (p < 0.05).

### Statement

Samples of palm sheath fiber were collected with the consent of the local producers. The authors guarantee that all procedures were carried out in compliance with the rules and regulations that applied.

## Results and discussion

### Characterization of the fabricated CN–ZnO bio-nanocomposites

#### Morphological investigation

**Figure 2:**
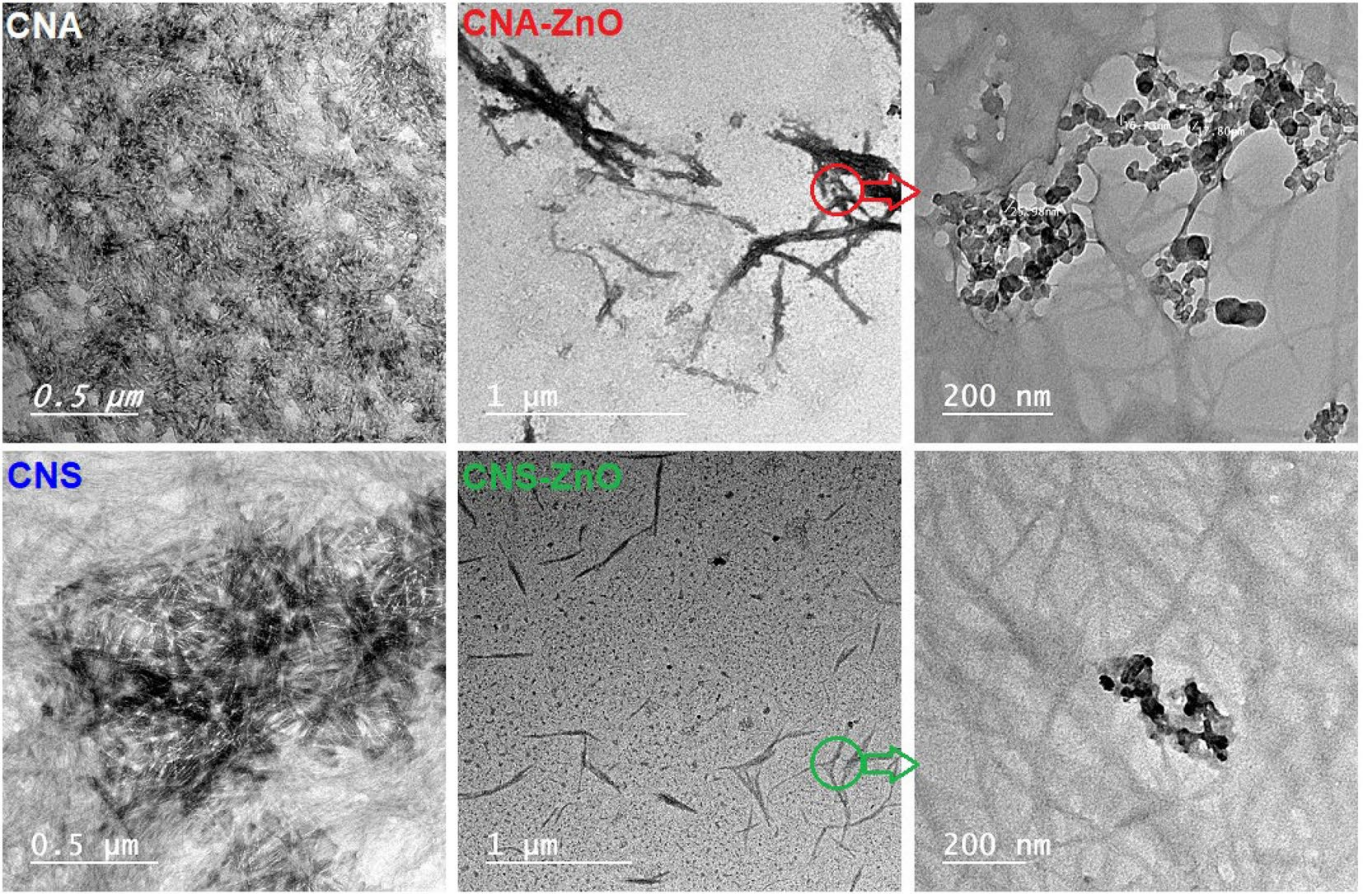
TEM micrographs of fabricated CNAs, CNSs, CNA–ZnO, and CNS–ZnO.

The effective fabrication of CNA–ZnO and CNS–ZnO nanomaterials using facile, efficient, and eco-friendly routes can be verified across assorted analytical tools. Figure 2 shows the TEM micrographs for the obtained CNAs, CNSs, CNA–ZnO, and CNS–ZnO nanomaterials. The prepared CNs exhibited uniform rod-like whiskers with narrow length distributions within 2.61–10.85 nm and 14.36–21.35 nm for CNAs and CNSs, respectively. Therefore, between the two extracted CNs, the CNAs exhibit smaller lengths when compared to the CNSs. Notably, the bio-nanocomposites samples, unlike the usual CN fibers, were not stained so that the inorganic NPs could be seen.

Moreover, Fig. 2 at low magnification shows the ZnO NPs contiguous to the CNA and CNS whiskers, whereas the higher magnification of CNA–ZnO and CNS–ZnO displays the relatively well dispersion of narrow-size ZnO metal oxide clusters inside the CNA and CNS fibers network particularly for the CNA–ZnO bio-nanocomposites. The size distribution of ZnO NPs depends clearly on the route used in their formation; thus, while CNA–ZnO has an average particle size in the range of 15.39–45.44 nm, the corresponding CNS–ZnO shows a smaller average size in the range of 15–30 nm.

**Figure 3:**
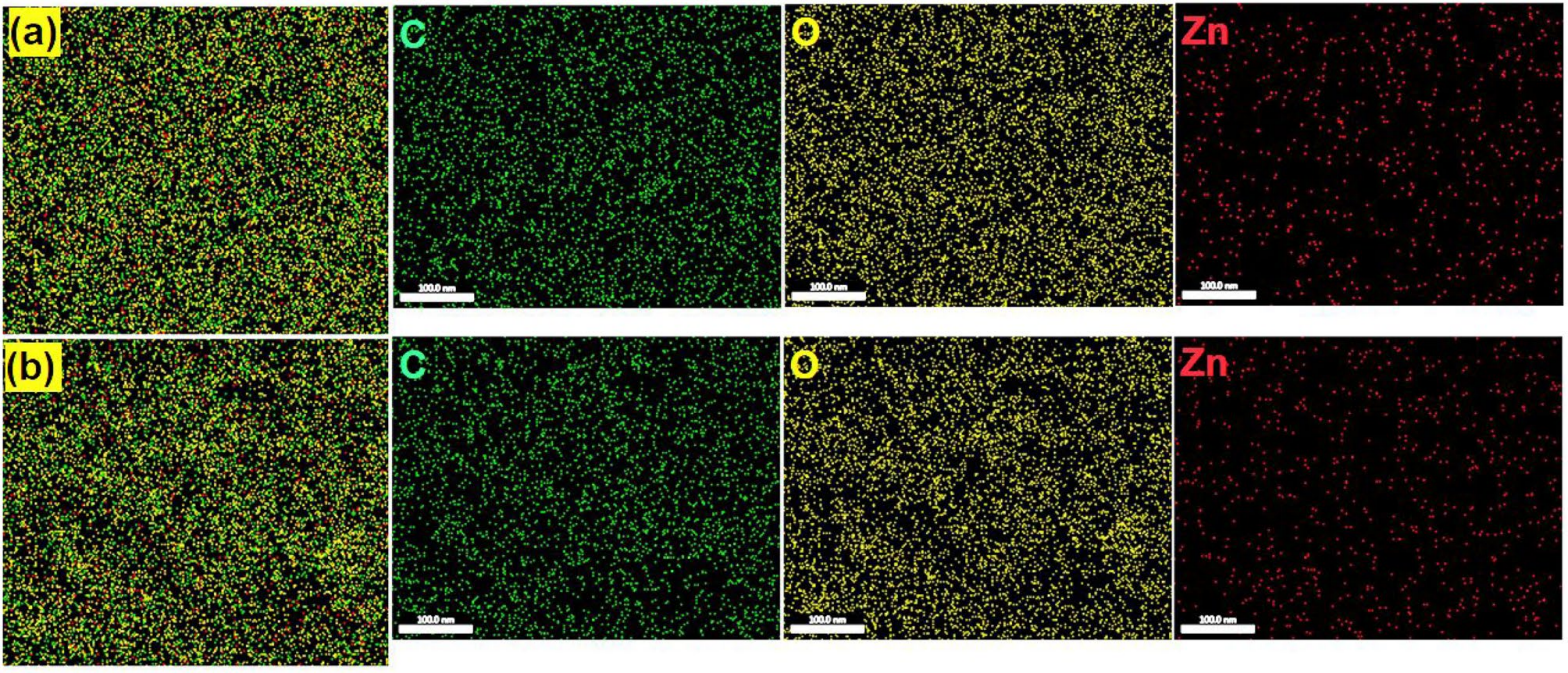
EDX Mapping of (**a**) CNA–ZnO and (**b**) CNS–ZnO bio-nanocomposites.

Table 3 and Fig. 3 show the composition analysis of the prepared bio-nanocomposites and their corresponding distribution maps using EDX spectroscopy. The recognition of Zn peaks in the EDX spectra indicates the successful intercalation of the ZnO NPs with the CNs (As can be seen in the Online Resource of the current study). Otherwise, the bands of C and O are ascribed to the binding energies of the CNs. Similarly, the EDX mapping of the prepared ZnO NPs shows the uniform distribution of Zn atoms along with C and O throughout the CN polymer matrix.

**Table 3:** EDX elemental analysis of CNA–ZnO, and CNS–ZnO bio-nanocomposites.

Sample	C K	O K	Zn K
	Weight % (at.%)		
CNA–ZnO	42.12 (50.52)	54 (48.63)	3.87 (0.85)
CNS–ZnO	41.7 (50.65)	52.78 (48.12)	5.51 (1.23)

#### FT-IR spectroscopy

**Figure 4:**
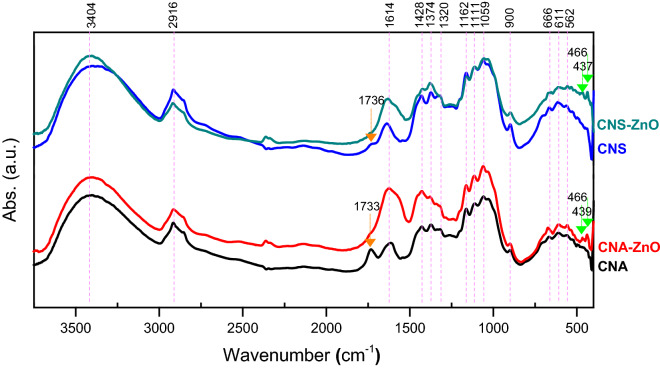
FT-IR spectra of fabricated CNAs, CNSs, CNA–ZnO, and CNS–ZnO.

The FT-IR spectra of the CNs extracted using APS (CNA) and sulfuric acid (CNS), along with the fabricated CNA–ZnO and CNS–ZnO bio-nanocomposites, are shown in Fig. 4. The spectral vibrations of these nanomaterials and their corresponding assignments are identified in Table 4. On the one hand, comparing the FT-IR spectra of CNA and CNS, bands at the wavenumbers of 1733 and 1736 cm^−1^, respectively (assigned to COO), can be observed, confirming that oxidation of C-6 and C–OH, terminal glucose rings, occurred during APS and sulfuric acid extraction of CNs ^4,14^. The degrees of oxidation of CNA and CNS, determined from the formula DO_IR_ = 0.01 + 0.7(*I*^1733^/*I*^1059^), are 0.373 and 0.210, respectively^64^. Moreover, the side reaction resulting from the extraction process activated the CNs with sulfate groups. This can be inferred by the appearance of the characteristic band ν S–O at a wavenumber of 611–614 cm^−1^^65^.

**Table 4:** FT-IR vibrational bands and their corresponding assignment for fabricated CNAs, CNSs, CNA–ZnO, and CNS–ZnO.

Band assignment	Band position (cm^−1^)			
	CNA	CNA–ZnO	CNS	CNS–ZnO
ν OH (inter and intra-molecular hydrogen bonds)	3404	3403	3403	3404
ν CH, ν_as_ CH_2_	2916	2914	2915	2920
ν COO	1733	–	1736	–
δ_s_ HOH	1614	1621	1637	1632
δ_ss_ CH_2_ of pyran ring, δ OH	1428	1428	1429	1429
δ_s_ CH, δ CH or ν CH_2_	1374	1379	1374	1379
ω CH_2_	–	1342	1320	–
δ CH	1262	1239	1277	1258
ν_as_ C–O–C from glucosidic units	1162	1161	1162	1158
ν_as_ Ring, ν C–C & C–O	1111	1113	1112	1111
ν C–O–C in β-(1 → 4) glucosidic linkage	1059	1060	1060	1056
ν C–O	1032	1032	1035	1035
γ C–H in cellulose ring due to β– linkage	900	900	899	898
ω or τ C–OH	666	672	665	666
ν S–O	611	608	614	611
ν M–O	–	466, 439	–	466, 437

*as* asymmetric, *s* symmetric, *ss* symmetric scissoring, *δ* bending, *γ* out of plane vibration, *ν* stretching vibration, *τ* twisting, *ω* wagging.

On the other hand, comparing the FT-IR spectra of the fabricated CNs with those of metal/metal oxide NPs, most of the bands appeared clearly with little change in their intensities and frequencies. These deviations in the intensities and frequencies of the absorption bands can be ascribed to the establishment of the metal oxides-polymer bonds^66^.

As displayed in Fig. 4, the wide absorption peak in the range between 3666 and 3000 cm^−1^, in all the fabricated nanomaterial spectra, is caused by the stretching vibrations of the OH groups (from absorbed H_2_O, secondary OH groups, and intramolecular and intermolecular H bonds). The bands at 2905–2920 cm^−1^ are linked to the aliphatic moieties and assigned to ν CH and ν_as_ CH_2_. The C–O–C from the glucosidic units and β-(1 → 4) glucosidic linkage displayed their characteristic bands at 1158–1163 and 1056–1061 cm^−1^, respectively. The inspection of the CNA–ZnO and CNS–ZnO FT-IR spectra shows that the disappearance of ν COO gives strong evidence of the metal oxides-polymer bonding process through the COO linkage. This inference is also supported by the appearance of new bands attributed to ν (M–O) in the far-infrared spectrum at 466 and 439 cm^−1^ and 466 and 437 cm^−1^ for CNA–ZnO and CNS–ZnO, respectively^36,66,67^.

#### XRD spectroscopy

**Figure 5:**
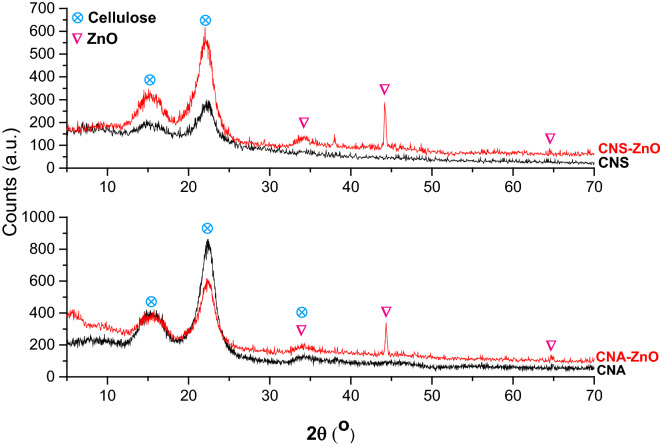
XRD profiles of fabricated CNAs, CNSs, CNA–ZnO, and CNS–ZnO.

The XRD diffractograms of CNA and CNS, presented in Fig. 5, reveal the diffraction peaks peculiar to allomorphic cellulose I at 2*θ* = 15.51° (1̅1̅0) and 22.28° (200) for CNA and 2*θ* = 15.02° (1̅1̅0) and 22.15° (200) for CNS. The data given in Table 5 show the interplanar spacing (*d*_200_*-spacing*, nm), crystallinity index (*CrI*, %), and mean crystallite size (*t*_200_, nm) as calculated by the Bragg, Segal, and Scherrer formulas, respectively. Table 5 shows that the prepared CNA–ZnO and CNS–ZnO bio-nanocomposites still retain their pristine lattice planes (Miller indices), which indicates the nontransformation or perturbation of the crystal core of the CN fibers during metal/metal oxide binding. However, there is a deviation in the Bragg angle of reflection from its original position. As we can see from the results, this shift depends on the route used in the preparation and the nanometal/metal oxide produced. Though the main diffraction peak of cellulose (200) shifts to a higher Bragg angle in CNA–ZnO, it shifts to a lower Bragg angle in CNS–ZnO. The mean crystallite size of the main cellulose lattice plane (200) was found to be affected in the same way as the Bragg angle. On the contrary, the crystallinity index is dependent on the type of CNs used in the preparation along with the preparation route. Moreover, CNA–ZnO, and CNS–ZnO show additional new diffraction peaks characteristic of ZnO at 34.1°, 39.4°, 44.22°, and 64.65° and 34.14°, 37.81°, 44.19°, and 64.52°, respectively^35^. These results imply that the intercalation of ZnO NPs into the CN polymer matrix did not alter the structural consistency of the matrix polymer but rather modified the molecular arrangement in the amorphous region of the polymer matrix. No peaks consistent with any used metal precursors were detected in the diffractogram, which supports the success of metal reduction under the applied reaction conditions.

**Table 5:** Bragg angel (2-theta), lattice plane (MI), crystallinity index (CrI), d_*200*_-spacing, mean crystallite sizes (t_*200*_) of CNs in the fabricated CNAs, CNSs, CNA–ZnO, and CNS–ZnO.

	2-theta, °			CrI, %	t_*200*_, nm	d_*200*_-spacing (calculated), nm
	(1̅1̅0)	(200)	(004)			
CNA	15.51	22.28	34.77	75.00	14.07	0.3987 (0.3983)
CNA–ZnO	15.55	22.31	34.10	60.91	15.41	0.3982 (0.3979)
CNS	15.02	22.15	–	53.75	14.01	0.4009 (0.4007)
CNS–ZnO	15.20	21.94	–	66.00	13.04	0.4047 (0.4045)

#### UV–Vis spectroscopy

**Figure 6:**
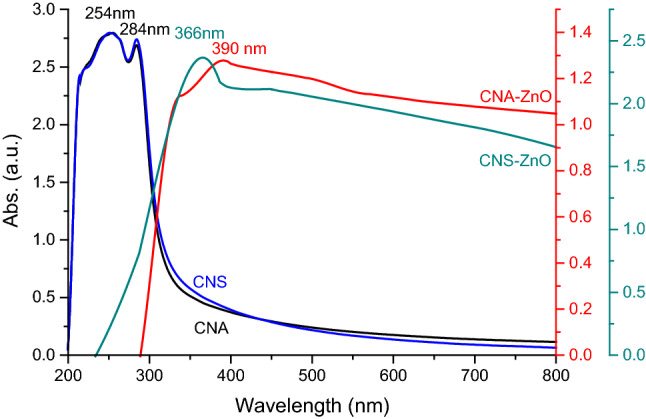
UV–Visible spectra of fabricated CNAs, CNSs, CNA–ZnO, and CNS–ZnO aqueous suspensions.

The optical characteristics of the fabricated CNAs, CNSs, CNA–ZnO, and CNS–ZnO suspensions were detected using UV–Vis absorption spectroscopy (Fig. 6). Significant absorbances were seen for the neat CNA and CNS solutions in the UV light range of 200 to 300 nm. Figure 6 shows two absorption peaks for the CNAs and CNSs at 254 and 284 nm. The carbohydrate structure of CNAs and CNSs, which can be seen as being made of hydrocarbon, ether, and alcohol groups linked by aliphatic bonds, is linked to the absorption at 254 nm^68^. Different theories from the literature were used to explain the literature's absorption peak at 284 nm in CNAs and CNSs. A 284 nm absorption peak in acid-treated and regenerated cellulose has been attributed to the carboxyl groups created during the treatment^69^, however other researchers have suggested that the acetal groups in the cellulose are responsible^70^. As well as APS, sulfuric acid can oxidize CNSs, resulting in the formation of a carboxyl group (see Fig. 4). Additionally, the CNAs and CNSs surface was treated with a sulfate ester group, and the sulfate ion displayed a comparable absorption peak at about 284 nm^71^. Therefore, the carboxyl and sulfate group coupled to CNA and CNS particles can be responsible for the absorption peak at 284 nm for the CNA and CNS suspensions. The successful preparation of the CN–ZnO bio-nanocomposites was confirmed by the appearance of a distinct UV–Vis absorption band at 390 and 366 nm for the CNA–ZnO and CNS–ZnO bio-nanocomposites, respectively, which is typical to the surface plasmon resonance of ZnO NPs^72^. Similarly, the absorption bands appear in the visible and ultraviolet regions, which are consistent with the bandgap of ZnO NPs at 289 and 236 nm for the CNA–ZnO and CNS–ZnO bio-nanocomposites, respectively^34,72^.

#### Zeta potential and particle size measurements

Zeta potential analysis was used to assess a crucial factor, the stability of CNA and CNS dispersions in aqueous conditions. CNA and CNS have measured zeta potentials of − 23 and − 36 mV, respectively. Hence, in neutral water, the prepared CNA and CNS suspensions had a negative zeta potential. Negatively charged carboxylate and sulfate groups on the surface of CNAs and CNSs are primarily responsible for their remarkable dispersion stability after APS and sulfuric acid treatments^4^. Even though, the zeta potential of CNAs and CNSs altered to − 13.1 and − 19.0 mV, respectively after ZnO NPs were bound. This can be explained by the surface amphoteric processes that, depending on the zero point of charge (ZPC) of the oxide, may affect an oxide or hydroxide surface when reacting with H^+^ or OH^−^ ions^73^. Since zinc oxide's ZPC ranges from 9 to 10, according to the literature, the hydroxide surfaces will absorb protons below the ZPC to form positively charged surfaces^74^. Consequently, the positive charge of ZnO NPs and the negative charge of CNAs and CNSs in the neutral medium have cumulated together to create the final charge of the bio-nanocomposites.

**Figure 7:**
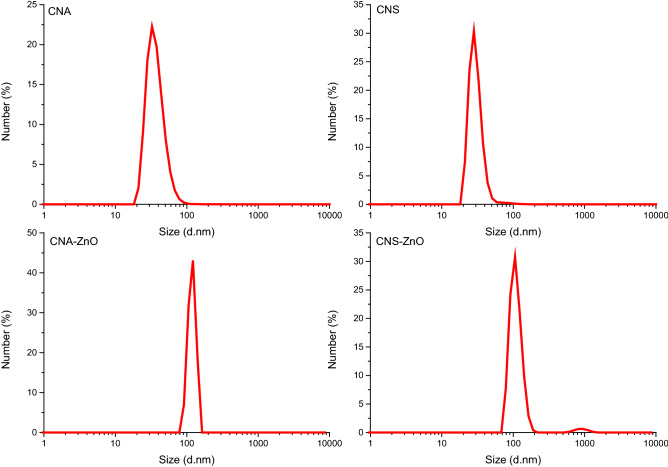
Size distribution by numbers from DLS study of the fabricated CNAs, CNSs, CNA–ZnO, and CNS–ZnO.

CNA and CNS particle size distributions show particles that are less than 100 nm in size. Particle sizes for CNAs and CNSs ranged from 21.04 to 122.42 nm. The average particle size and width for CNAs were 37.39 nm and 15.37 nm, respectively. While CNSs have an average particle size and breadth of 30.36 nm and 8.81 nm, respectively. Thus, CNS specimens had a smaller particle size distribution than the CNA (Fig. 7). The results presented allows us to conclude that the APS and sulfuric acid treatments we carried out were sufficient for generating CNs. However, CNA–ZnO and CNS–ZnO bio-nanocomposites have larger average particle sizes, measuring 118.43 nm and 109.5 nm, respectively. This may be because of several factors, the most significant of which is the lower zeta potential, which in turn may cause a rapid accumulation of the particles. It may also be because of the chelating effect brought on by the presence of ZnO NPs between the CNA and CNS fibers. Furthermore, due to possible interference, we are unable to distinguish between the average particle sizes of CNs and ZnO NPs.

#### Mechanism of in situ ZnO NPs synthesis by NCs

From the results obtained for TEM, EDX, and UV–Vis analysis, we can conclude the direct effect of reaction temperature on the particle size and concentration of ZnO NPs prepared using CNs as both reducing agents and stabilizers. Thus, the higher the temperature is, the larger the particle size and the lower their concentration will be. In addition, we can observe that as ZnO concentrations decrease, their diameters increase. This may be similar to what happens when NPs are synthesized via a solid–vapor mechanism. Therefore, a lower ZnO content means that fewer nuclei were molded and thus the entering Zn^2+^ ions would be added to the diameter of the ZnO NPs, expanding them. Whereas, a higher ZnO content means that more nuclei were formed that grow at a faster rate and therefore have less chance of growing along the diameter. This was also predicted when carboxymethyl chitosan was used to prepare ZnO NPs at different temperatures and when a ZnO nanowire was immobilized on the surface of cellulose fibers using a single-step hydrothermal process^72,75^.

The proposed mechanism for the growth of these NPs depends mainly on the eminent model provided by CNs as a reducing and stabilizing agent, which originate primarily as a result of APS and sulfuric acid treatments of unbleached and bleached fibers, respectively. The mode of action of the APS treatment involves in one step the removal of lignin, hemicellulose, pectin, and other plant contents through formed SO_4_^−^ free radicals and H_2_O_2_ at high temperature. These free radicals and H_2_O_2_ can collectively infiltrate and disintegrate the cellulosic amorphous regions to form CNs and break down the aromatic rings of lignin to bleach the pulp^76^. However, sulfuric acid treatment requires the pretreatments of the unbleached pulp with bleaching agents to remove other fiber contents (e.g., hemicellulose and lignin), which consequently affects cellulose crystallinity^14^. The hydrolysis of sulfuric acid is therefore a heterogeneous process consisting of the penetration of the acid into the cellulose fibers, pursued by the dissolution of glycoside bonds^77^. Primarily, the amorphous regions of cellulose fibers are attacked by the acid, as they are the most easily accessible and have the greatest surface area, followed by the regions of higher crystallinity. A side reaction can ensue between surface hydroxyl groups of cellulose fibers and the sulfuric acid, forming surface-charged sulfate esters that assist in improving the diffusion of CNs in the polar solvents^78^.

CNs, extracted from PSFs, could be an eminent model for metal/metal oxide NP green fabrication because of their efficiency to suppress the growth of NPs. This power ensues from the intramolecular and intermolecular hydrogen bonding structure in CNs. Additionally, the CN extraction procedures produce additional ionic moieties (namely, COO^−^ and SO_4_^–^) as well as three reducing hydroxyl groups per anhydroglucose unit; as a result, CNs have anchor points for the attachment of metal/metal oxide NPs. Using the conductometric titration method as specified in the standard protocol (SCAN-CM65:02), the total charge density of carboxylate and sulfate groups on CNA and CNS surfaces was determined to be 1370 and 1430 mol/g, respectively.

Based on this, the CN mode of action in the in situ synthesis of ZnO NPs can be proposed as follows; chelating interactions occur between Zn^+2^ ions and the active moieties and hydroxyl groups on the surface of the cellulose fibers, forming a Zn–O–X bond. Further, these metal atoms act as nucleation centers and continue to grow in one dimension. This process continues until larger high-nuclear-energy molecules are formed, which are stabilized and terminated by interaction with the cellulose polymer^75,79^. Thermal drying at 60 °C for 6 h yields the CN–ZnO bio-nanocomposites' final product. This was inferred by noticing ZnO NPs stuck between the fibers of CNs, as shown in the TEM micrographs (Fig. 2), and the uniform distribution of Zn alongside carbon and oxygen, as seen in the EDX mapping (Fig. 3).

### Antimicrobial activity

#### Bactericidal effect of the synthesized CN–ZnO bio-nanocomposites on tested strains

**Figure 8:**
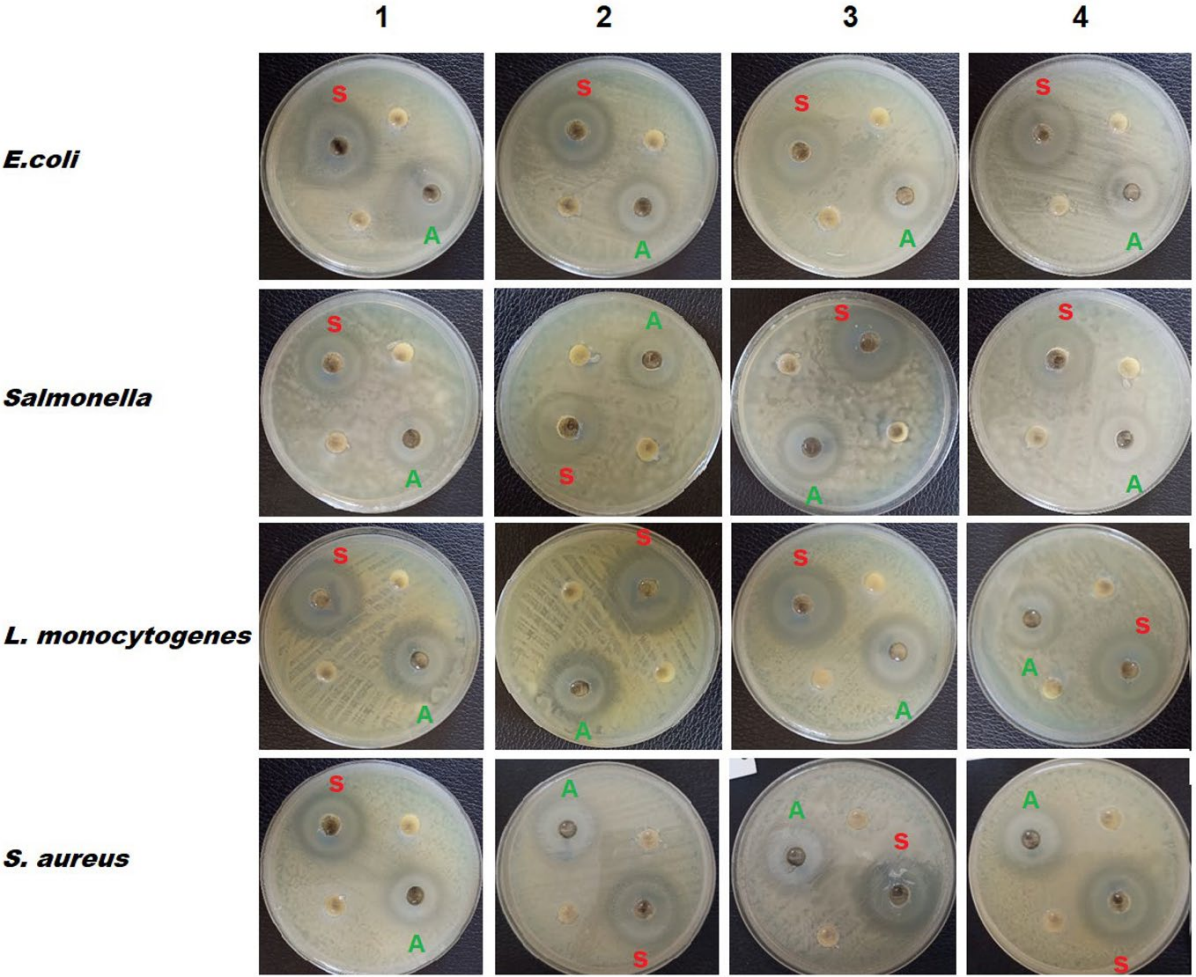
Antibacterial activity test shows the inhibition zone of tested strains caused by (A) CNA–ZnO and (S) CNS–ZnO bio-nanocomposites suspensions [Injected samples that do not display any region of inhibition are representative of the CNAs and CNSs].

In the antibacterial activity test, the pure CNs (CNA and CNS) suspensions were selected as the control. No antibacterial activity was observed for both of them. In contrast, the inclusion of ZnO NPs on the surface of CN whiskers induced the antibacterial activity with which the suspension of CN–ZnO bio-nanocomposites showed significant inhibition of bacterial growth. All the tested isolates were inhibited by both suspensions (CNA–ZnO and CNS–ZnO), but the CNS–ZnO suspension showed a higher inhibition zone for all the tested isolates. Divergence in the extent of the inhibition area among the diverse groups of bacteria can be relatively disclosed. On the one hand, the highest inhibition zone was observed for *L. monocytogenes*, which ranged from 26 to 31 mm in the case of CNA–ZnO and 28 to 35 mm in the case of CNS–ZnO. On the other hand, the minimum inhibition zone was detected for *Salmonella*, which ranged from 22 to 25 mm in the case of CNA–ZnO and 25 to 28 mm in the case of CNS–ZnO, as shown in Table 6 and Fig. 8. Our results are following those of Zhao et al. who found that a pure CNs suspension did not have any antibacterial activity^80^.

**Table 6:** Antibacterial activity of CNs (CNA & CNS), CNA–ZnO, and CNS–ZnO suspension against *E. coli*, *Salmonella* spp., *L. monocytogenes,* and *S. aureus* strains.

Bacterial strains	Diameter of inhibition zone (mm)		
	CNs	CNA–ZnO	CNS–ZnO
***E. coli***			
1	0	25 ± 0.08	27 ± 0.20
2	0	24 ± 0.0.6	26 ± 0.04
3	0	24 ± 0.16	26 ± 0.29
4	0	24 ± 0.04	28 ± 0.08
***Salmonella***			
1	0	22 ± 0.05	25 ± 0.09
2	0	23 ± 0.04	25 ± 0.23
3	0	25 ± 0.13	27 ± 0.09
4	0	24 ± 0.20	28 ± 0.05
***L. monocytogenes***			
1	0	28 ± 0.04	31 ± 0.08
2	0	29 ± 0.16	30 ± 0.0.9
3	0	31 ± 0.04	35 ± 0.0.16
4	0	26 ± 0.04	28 ± 0.14
***S. aureus***			
*1*	0	24 ± 0.16	26 ± 0.07
2	0	26 ± 0.12	30 ± 0.19
3	0	25 ± 0.07	27 ± 0.05
4	0	22 ± 0.17	27 ± 0.12

The mode of action of ZnO NPs against bacteria includes the release of metal ions that penetrate the bacterial cell membranes, interfering with functional groups in nucleic acid and protein, thereby inhibiting the activity of enzymes. Accordingly, the change in the cell structure will eventually lead to the inhibition of microorganisms^44^. Furthermore, another potential mode of action that could be exerted by ZnO NPs involves the release of the active form of oxygen, which initiates electrostatic binding, leading to modification of DNA or enzyme pathways as well as the prokaryotic cell wall^38^. This oxidative stress weakens bacteria's mitochondria, DNA, and membranes, resulting in bacterial death^81^. Accordingly, the foremost impact in inhibiting bacterial growth, as it was suggested by many researchers, could be attributed to the creation of H_2_O_2_ on the surface of ZnO NPs^82^.

##### The MIC and MBC of the synthesized CN–ZnO bio-nanocomposites

The antibacterial effects of the prepared CN–ZnO bio-nanocomposites were also assessed by employing the MIC procedure. This technique was implemented to demonstrate the lowest concentration of the fabricated CN–ZnO bio-nanocomposites that can stop or hinder bacterial growth. From the studies conducted on the 96-well titer plate, the minimum concentration of CNA–ZnO and CNS–ZnO suspensions to fully hinder the growth of *Salmonella*, *L. monocytogenes*, *E. coli*, and *S. aureus* is ranging between 0.25 and 1 μg/mL.

On the one hand, the MIC values of CNS–ZnO (0.5–1 μg/mL) against the *E. coli* and *L. monocytogenes* strains were higher than those of CNA–ZnO (0.25–1 μg/mL). On the other hand, the MIC values of CNA–ZnO and CNS–ZnO against *Salmonella* and *S. aureus* were in the range of 0.25–1 μg/mL. Furthermore, CNA–ZnO, and CNS–ZnO showed effective antibacterial agents (MBCs of 0.5–2 μg/mL), as shown in Table 7. This indicates that CNS–ZnO influences a very low concentration.

**Table 7:** SIC, MIC, and MBC of CNA–ZnO and CNS–ZnO against *E. coli, Salmonella spp, L. monocytogenes, and S. aureus* strains.

Bacterial strains	Concentration of SIC, MIC, MBC (µg/ml)					
	CNA–ZnO			CNS–ZnO		
	SIC	MIC	MBC	SIC	MIC	MBC
***E. coli***						
1	0.5	1	2	0.5	1	2
2	0.25	0.5	1	0.5	1	2
3	0.125	0.25	0.5	0.5	1	2
4	0.125	0.25	0.5	0.25	0.5	1
***Salmonella***						
1	0.5	1	2	0.25	0.5	1
2	0.25	0.5	1	0.5	1	2
3	0.125	0.25	0.5	0.125	0.25	0.5
4	0.25	0.5	1	0.5	1	2
***L. monocytogenes***						
1	0.5	1	2	0.5	1	2
2	0.125	0.25	0.5	0.5	1	2
3	0.25	0.5	1	0.25	0.5	1
4	0.5	1	2	0.5	1	2
***S. aureus***						
1	0.5	1	2	0.125	0.25	0.5
2	0.25	0.5	1	0.125	0.25	0.5
3	0.125	0.25	0.5	0.5	1	2
4	0.125	0.25	0.5	0.125	0.25	0.5

The high surface/volume ratio of these nanostructured compounds, which provides a broader contact area with agents in the environment, is thought to be the main cause of their antibacterial activities. The capacity to smoothly penetrate cell membranes disorders several intracellular processes, leading to reactivity and antimicrobial activity^83^. So, the broth dilution assay is an accurate and reliable approach for determining the antibacterial activity of examined nanoparticles^84^.

### Detection of toxin genes in all the tested strains via conventional PCR

All the tested isolates carried the toxin genes and gave characteristic bands except *S. aureus* which harbored *sea* and *seb* genes only. Approximately 95% of food poisoning cases by staphylococcal are related to the classical *SEs* (*SEA to SEE*)^85^. Staphylococcal enterotoxins stimulate T-cell induction causing systemic diseases including toxic shock syndrome^86^.

According to our study, *E. coli* strains carried Shiga toxins *Stx-1* and *Stx-2*. These outcomes are in accordance with those of Reyna et al. who proved that *E. coli* strains form Shiga toxins *Stx-1* and *Stx-2* that participate in their virulence. Shiga-toxin production is the main cause of hemolytic uremic and hemorrhagic colitis syndrome, specifically in the elderly and infants. Further, virulent *E. coli* O157:H7 is categorized by the existence of *Stx-1* and *Stx-2*^87^.

Moreover, the enterotoxin (*stn*) gene was detected for the *Salmonella* strains. Similar results were obtained by Prager et al. who demonstrated the *stn* gene as a suitable PCR target for detecting *Salmonella*^88^. Pathogenicity of *Salmonella* strains has been associated with several virulence genes found in islets of chromosomally pathogenic *Salmonella*^89^. Another chromosomal gene like *stn* codes for the formation of enterotoxin which is an originating agent of diarrhea^90^.

### Quantitative assessment effect of the tested bio-nanocomposites extracts on toxin virulence genes in all bacterial strains via qRT-PCR

Using RT-PCR, the amounts of testing virulence gene products (cDNA) before and after treatment with a SIC of the tested CN–ZnO bio-nanocomposites can be compared as qRT-PCR has emerged as one of the most used approaches for gene expression analysis due to its benefits of great sensitivity and specificity^91^.

**Figure 9:**
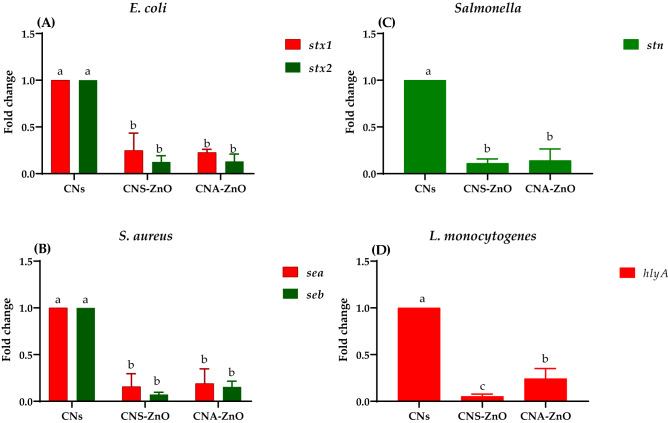
The relative mRNA expression levels of genes related to toxins through tested bacterial strains before and after treatment with CNs (CNA and CNS), CNA–ZnO, and CNS–ZnO.

The fold changes in the *stx1* and *stx2* gene expression after treatment with SIC of the CNA–ZnO suspension were (0.099: 0.381-fold) and (0.0165: 0.217-fold), respectively, which were close to the fold changes in the same gene expression after treatment with SIC of CNS–ZnO, which were (0.073: 0.441-fold) and (0.010: 0.211-fold), respectively (Fig. 9a). These results may be driven from the expression of certain host immune genes^92^ as a direct response to the effective treatment.

Regarding *S. aureus*, the downgrading of the toxin *sea* and *seb* gene expression after treatment with the SIC of the CNA–ZnO suspension was expressed by the fold changes of (0.014:0.368 fold) and (0.006:0.510 fold), respectively, which were higher than the fold changes of the CNS–ZnO of (0.007:0.293 fold) and (0.004:0.101 fold), respectively (Fig. 9b). These remarks could also be related to *agr*-dependent creation of these SEs^93^, as CNA–ZnO indeed has a strong inhibitory influence on *agr*-expression.

The *stn* gene of *Salmonella* was downregulated by CNS–ZnO (0.045:0.143 fold), whereas CNA–ZnO showed a fold change of (0.059:0.489 fold) (Fig. 9c). Consequently, this could facilitate the passing of *Salmonella* from the apical area to the basolateral area, thus terminating the *Salmonella* vicious cycle with an improvement of its virulence^94^.

Regarding *L. monocytogenes*, there was a strong downregulation in the *hlyA* gene in the CNS–ZnO group (0.027:0.076 fold), whereas CNA–ZnO showed a fold change of (0.124:0.392) (Fig. 9d). Pieta et al. observed similar results but for natural substances existing in the essential oil of *Baccharis psiadioides*, as the virulence genes of *L. monocytogenes*, such as *prfA*, *fur*, *hlyA*, *actA*, and *agrA*, were downregulated (*p* < 0.05)^95^.

Several studies mentioned the molecular action of ZnO NPs on several multidrug-resistant bacteria. Xie et al. studied a considerable group of genes of *Campylobacter jejuni* that participated in toxin production, pathogenesis, motility, and cell stress response. Reverse transcription-quantitative PCR indicated that, in response to remediation with ZnO NPs, the expression levels of the general stress response gene (*dnaK*) and two oxidative stress genes (*ahpC* and *katA*) were improved 17, 7, and 52-fold, respectively^96^. Also, Nejabatdoust et al. proved that synthesized ZnO NPs by glutamic acid with an approximate size of 100–30 nm could alter the expression of *NorA* gene which evolved in efflux pump formation in multi-drug resistant *S. aureus* isolates^97^.

Further, Abdelraheem and Mohamed found that ZnO NPs remarkably downregulated the expression levels of all virulence genes and the biofilm of *Pseudomonas aeruginosa* clinical isolate other than the *toxA* gene, which was upregulated. The fold change decreases of the quorum sensing genes *pqsR, rhl0049*, and *LasR* after treatment via ZnO NP were 8.7, 6.3, and 10.4-fold (*p-value* < 0.0001), respectively. Likewise, ZnO NPs downregulated other genes accountable for biofilm creation, i.e., the *PelA* and *LecA* genes by 5.6 and 4.7-fold (*p-value* < 0.0004), respectively. Also, ZnO NPs downregulated virulence genes *lasA* and *exoS* by 5.2 and 3.7-fold, respectively (*p-value* < 0.008). In addition, the *toxA* gene was downregulated by 1.9-fold (p-value = 0.37) after ZnO NPs remediation, thus no statistically considerable upregulation was recorded^98^.

In the current study, all tested virulence genes were remarkably down-regulated, implying a regression of bacterial pathogenicity after remedy, especially in the CNS–ZnO group. To the best of our knowledge, the present study is the first prevalence of demonstrating the influence of CNs and CN–ZnO bio-nanocomposites on levels of gene expression in toxigenic bacteria in Egypt and the Middle East.

## Conclusion

The use of cellulose for material fabrication and its applications has received scientific and technological interest owing to its distinct usage direction in various forms of preparation. In particular, CNs show superior properties compared to their bulk materials. The strong intramolecular and intermolecular hydrogen bonding that exists in CN fibers along with active moieties (carboxyl and sulfate groups) and abundant free hydroxyl groups creates a three-dimensional mold structure for the fabrication of metal/metal oxide NPs. The two extracted CNA and CNS have different morphological characters, which, in turn, form distinct CN–ZnO bio-nanocomposites. Further to the obtained results, the direct effect of the reaction temperature and properties of CNs molds on the particle size and concentration of ZnO prepared by the sono-co-precipitation method can be inferred. Thus, the size and concentration of fabricated ZnO NPs are inversely proportional to each other and depend on the type of CNs used and the applied reaction temperature.

The current study emphasizes the dual-target therapy of novel CN–ZnO bio-nanocomposites, which enhances bacterial treatment by preventing in vitro bacterial growth and focusing on bacterial toxin synthesis, thereby reducing the emergence of antibiotic resistance. The anti-toxigenic properties are likely due to the direct binding and downregulation of toxin-associated genes of tested Gram-positive and Gram-negative bacteria. According to the aforementioned results, the synthesized CNS–ZnO bio-nanocomposite offer intriguing characteristics that render them excellent alternative products for targeted drug delivery, controlling microorganism’s reproduction and toxins in food.

## Supplementary Information

Supplementary Information.

## Data Availability

All data generated or analysed during this study are included in this published article and its Supplementary Information files. The ATCC bacterial strains tested in this study are obtained from Thermo Fisher Specialty Diagnostics Ltd, Hampshire, UK, and available at https://thermofisher.com/microbiology.
