# Supplementary material for: Green synthesis of cellulose nanocrystal/ZnO bio-nanocomposites exerting antibacterial activity and downregulating virulence toxigenic genes of food-poisoning bacteria
Source: Sci Rep. 2022 Oct 7;12:16848. doi: 10.1038/s41598-022-21087-6 (PMC9547054; doi:10.1038/s41598-022-21087-6)
Supplement: Supplementary file 1 — Supplementary Information. [file 41598_2022_21087_MOESM1_ESM.docx]

**Green Synthesis of Cellulose Nanocrystal/ZnO Bio-nanocomposites Exerting Antibacterial Activity and Downregulating Virulence Toxigenic Genes of Food-Poisoning Bacteria**

Ghada E. Dawwam^1^, Mona T. Al-Shemy^2*^, Azza S. El-Demerdash^3^

^1^ Botany and Microbiology Department, Faculty of Science, Benha University, Benha, Egypt

^2^ National Research Center, Cellulose and Paper Department, 33 El-Bohouth St. (Former El-Tahrir St.), Dokki, Giza, Egypt. P.O. 12622

^3^ Agriculture Research Center (ARC), Animal Health Research Institute (AHRI), Zagazig, Egypt

*Corresponding author: Mona T. Al-Shemy. E-mail: [mt.el-shemy@nrc.sci.eg](mailto:mt.el-shemy@nrc.sci.eg). Orcid ID: 0000-0001-9290-5955. Tel: +2-01154456633. El Buhouth St., Dokki, Cairo, Egypt Postal Code 12622


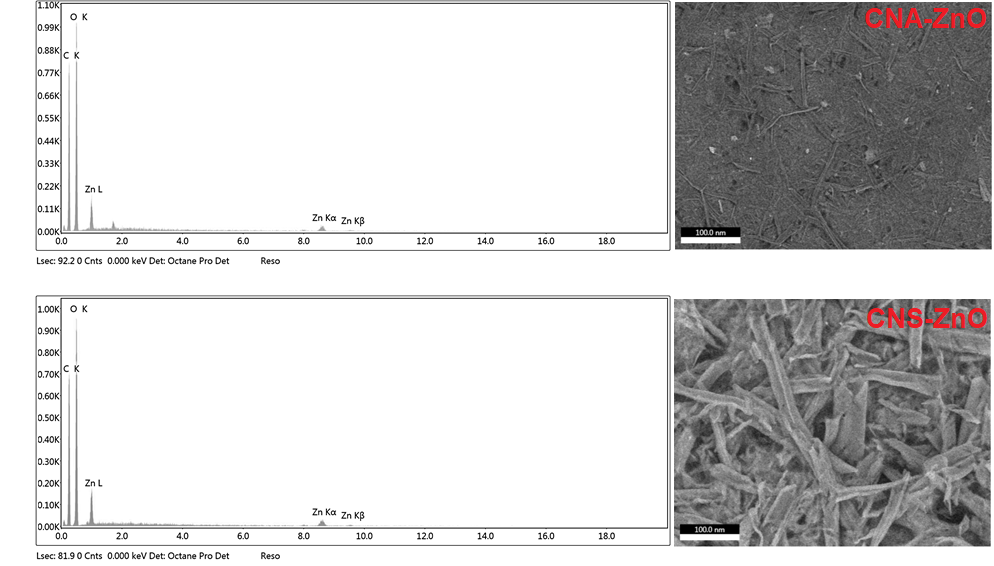


EDX and SEM analysis of fabricated materials
